# Supplementary figures and images for: A novel antibody for the detection of alternatively spliced secreted KLOTHO isoform in human plasma
Source: PLoS One. 2021 Jan 22;16(1):e0245614. doi: 10.1371/journal.pone.0245614 (PMC7822350; doi:10.1371/journal.pone.0245614)

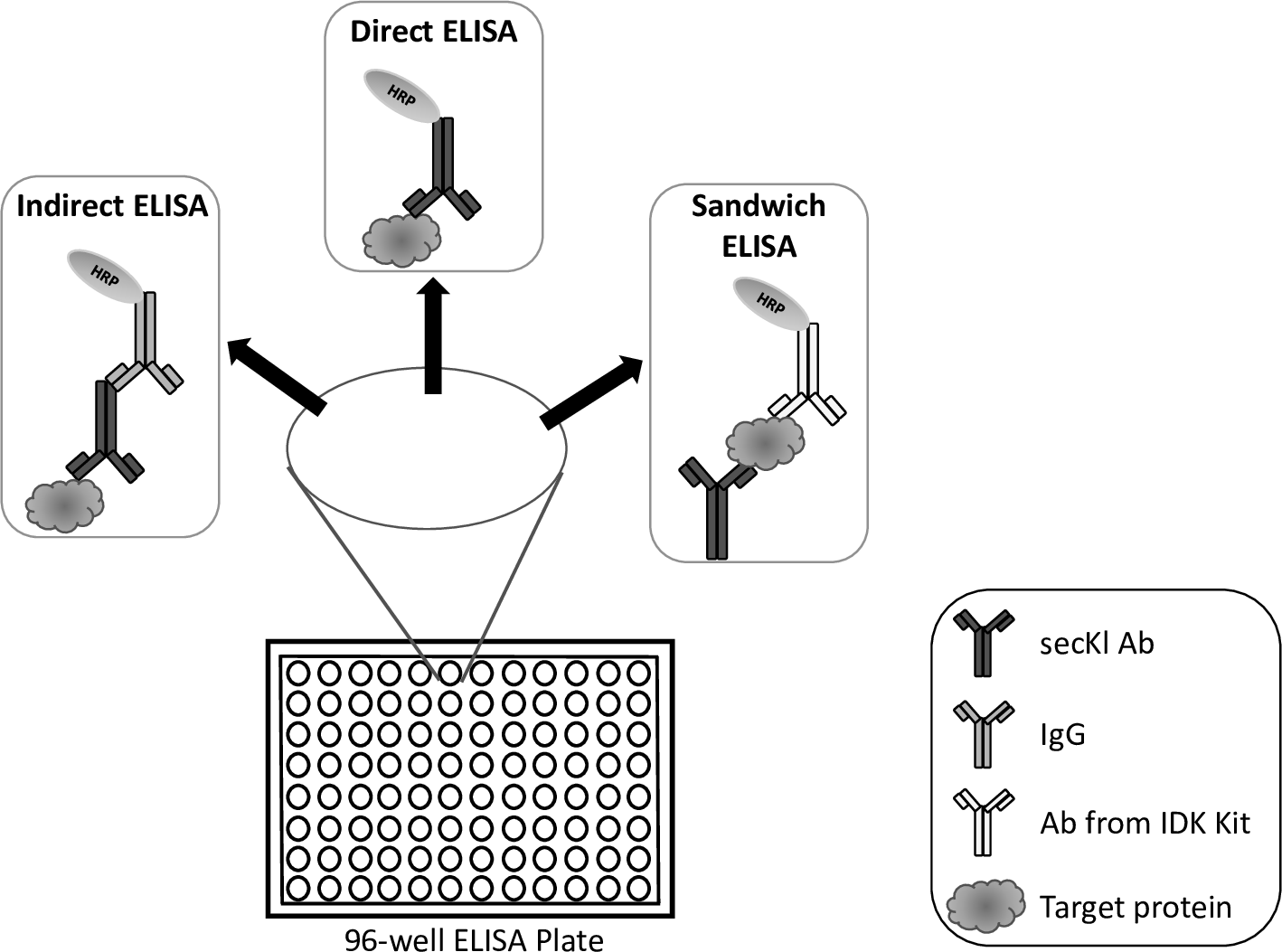

Supplement: S1 Fig — In the direct and indirect ELISA assay, secKL protein was coated on the well and either labelled secKL Ab (direct) or a labelled secondary Ab (indirect), in combination with the secKL Ab, was used to detect the signal. In the sandwich assay, secKL Ab was coated on the well and served as a capture Ab and labelled Ab from the IDK Kit was used as the detection Ab. (TIF) [file pone.0245614.s001.tif]

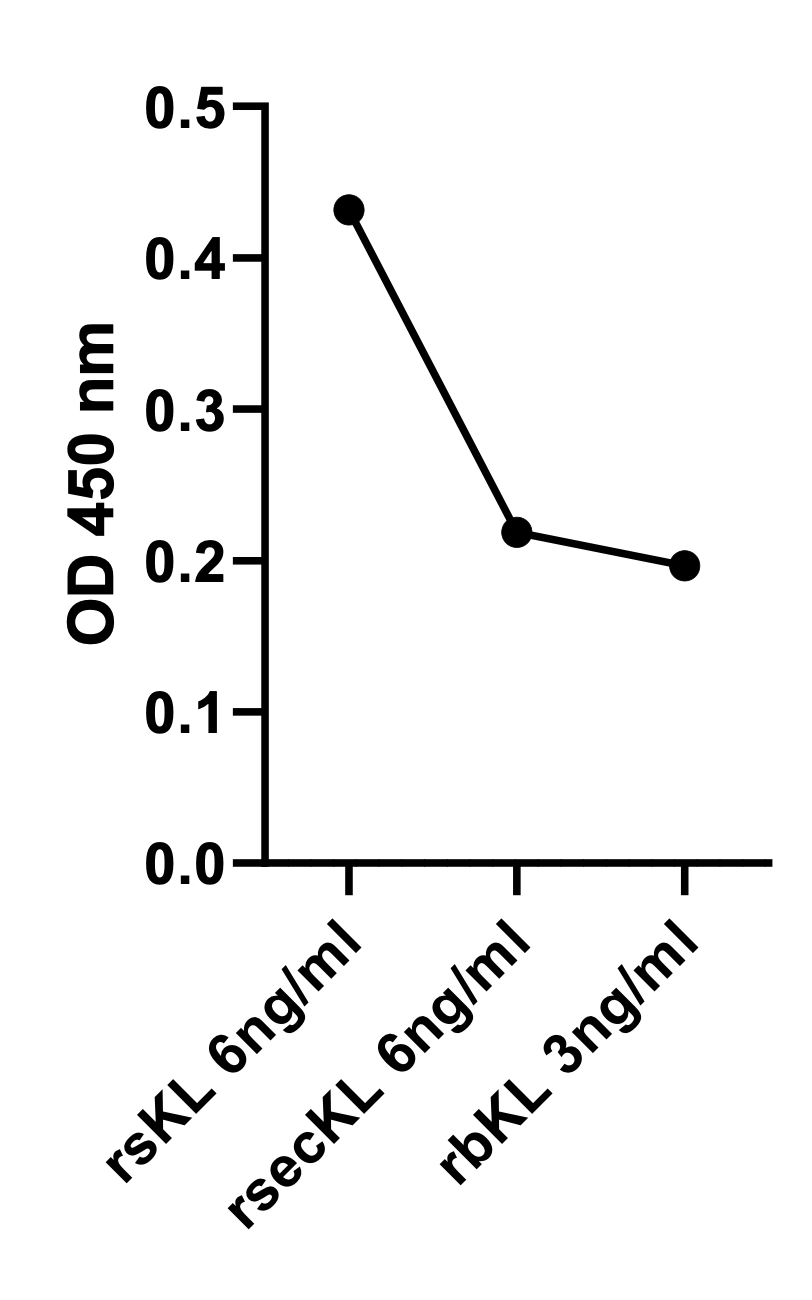

Supplement: S2 Fig — (TIF) [file pone.0245614.s002.tif]

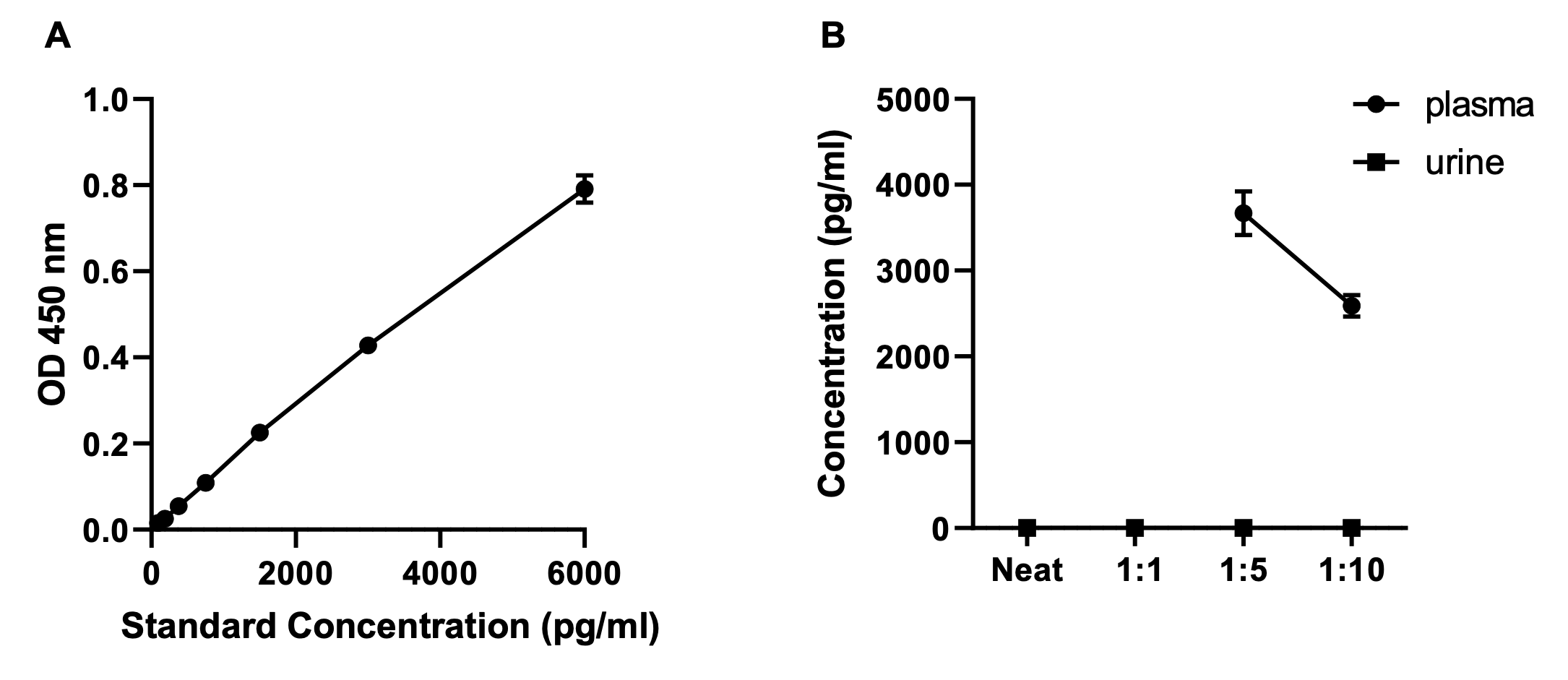

Supplement: S3 Fig — (A) standard curve with secKL protein concentrations of 6000pg/ml, 3000pg/ml, 1500pg/ml, 750pg/ml, 375pg/ml, 187.5pg/ml, 93.75pg/ml (B) seckL protein concentrations in human plasma and urine samples at indicated dilutions. While there is linear correlation in the plasma measurement, no protein detection observed in the urine, indicating that the Ab is able to detect differential expression in different specimens. (TIF) [file pone.0245614.s003.tif]

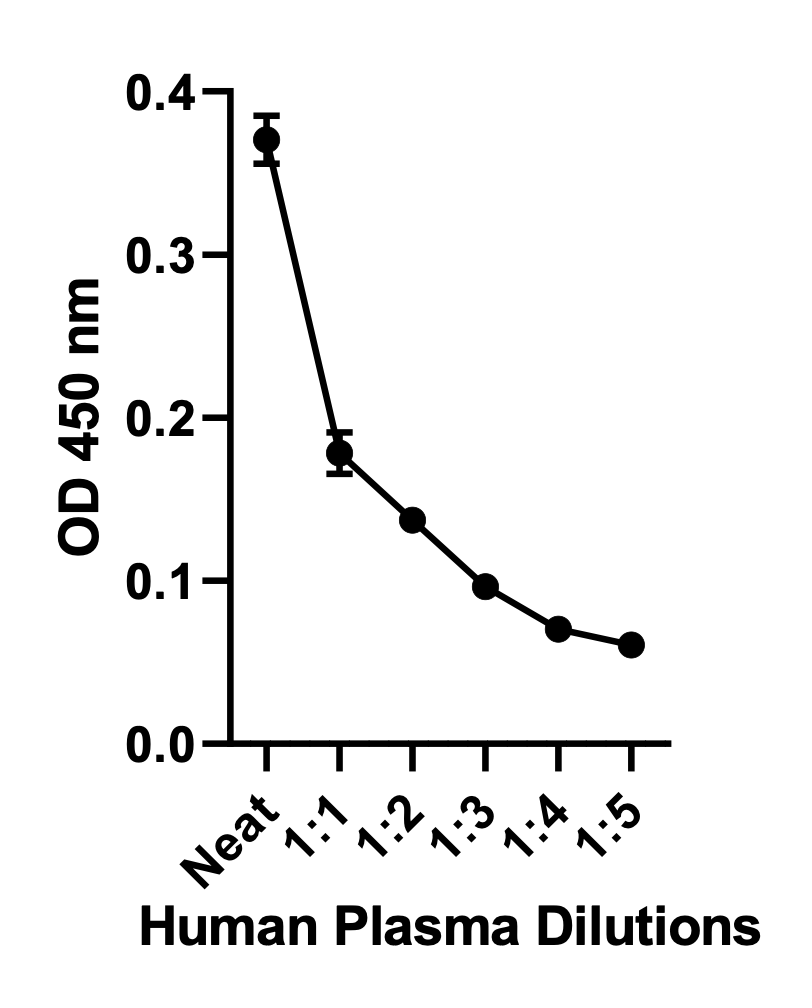

Supplement: S4 Fig — (TIF) [file pone.0245614.s004.tif]
