# Supplementary material for: A novel antibody for the detection of alternatively spliced secreted KLOTHO isoform in human plasma
Source: PLoS One. 2021 Jan 22;16(1):e0245614. doi: 10.1371/journal.pone.0245614 (PMC7822350; doi:10.1371/journal.pone.0245614)
Supplement: S1 Table — (DOCX) [file pone.0245614.s005.docx]

S1 table

| Sample | Race | Gender | Age | SCr | eGFR |
| --- | --- | --- | --- | --- | --- |
| CKD1 | White | Male | 71 | 3.41 | 18 |
| CKD2 | Unknown / Not Reported | Female | 54 | 1.49 | 36 |
| CKD3 | White | Female | 37 | 1.02 | NA |
| CKD4 | White | Female | 52 | 2.64 | 19 |
| Normal 1 | Black or African American | Male | 32 | 1.13 | NA |
| Normal 2 | White | Male | 42 | 0.97 | NA |
| Normal 3 | White | Female | 38 | 0.78 | NA |
| Normal 4 | White | Male | 29 |  | NA |
| Normal 5 | White | Female | 58 | 0.77 | NA |

Kidney function parameters for plasma samples

NA: not available; Scr: Serum Creatinine; eGFR: estimated Glomerular Filtration Rate
