# Supplementary material for: A novel antibody for the detection of alternatively spliced secreted KLOTHO isoform in human plasma
Source: PLoS One. 2021 Jan 22;16(1):e0245614. doi: 10.1371/journal.pone.0245614 (PMC7822350; doi:10.1371/journal.pone.0245614)
Supplement: Attachment — Submitted filename: Response to reviewers.pdf [file pone.0245614.s006.pdf]

Dear Dr. Ping-Hsun Wu,

Thank you for your letter dated October 21, 2020 with comments. We greatly appreciate and agree with yours and the reviewers' suggestions, and we have aimed to address all the comments in this letter and incorporated all suggestions and points into the paper. The changes have been highlighted in the revised manuscript for ease of reference.

We hope that you will find favor in all the information provided. We would like to express our gratitude for your consideration of our manuscript and we look forward to hearing from you.

Thank you very much for your time and consideration.

Yours sincerely,

Li-Li Hsiao, MD, PhD, FACP
Director, Asian Renal Clinic
Director, Global Kidney Health Innovation Center
Co-Program Director, Harvard Summer Research Program in Kidney Medicine
Brigham and Women's Hospital
Harvard Medical School
BLI Room 443, 221 Longwood Ave.
Boston, MA. 02115
617-525-7366 (o)
Phone: (617) 525-7366
Fax: (617) 525-7386
E-mail: lhsiao@bwh.harvard.edu

Point-by-point response to the editor's and reviewers' comments

Journal Requirements:

When submitting your revision, we need you to address these additional requirements.

1. Please ensure that your manuscript meets PLOS ONE's style requirements, including those for file naming. The PLOS ONE style templates can be found at

https://journals.plos.org/plosone/s/file?id=wjVg/PLOSOne_formatting_sample_main_body.pdf and https://journals.plos.org/plosone/s/file?id=ba62/PLOSOne_formatting_sample_title_authors_affiliations.pdf

We thank the editor's for pointing out our misstep. We have modified the manuscript following the recommended guidelines.

2. In the Methods section, please provide the location of the GenScript company that synthesized the peptides for antibody generation.

Per editor's suggestion, we have updated this information in the "Materials and Methods" section Line 73-74, page 5

"Following peptide sequence was provided to GenScript (GenScript USA Inc. 860 Centennial Ave. Piscataway, NJ 08854) for antibody generation: SQLTKPISSLTKPYH"

Additional Editor Comments (if provided):

Re-plot the figures with consistent scales were advised, including Fig 1A, 1B, 1C, 1D, 1E, 1F, and 2A. The numbers of experimental replicates were recommended to demonstrate in the method section. Please revise as the reviewers' suggestions.

We thank you for your comments. We have re-plotted all graphs to maintain consistency in terms of the scales. In addition, each result shown in this manuscript is a representative of three replicates. This information has also been updated in the "Materials and Methods", Line 85-87, page 5.

"Each result is a representative of three experimental replicates. Error bars are +/- standard deviation unless indicated otherwise."

Reviewers' comments:

Reviewer's Responses to Questions

Comments to the Author

1. Is the manuscript technically sound, and do the data support the conclusions?
The manuscript must describe a technically sound piece of scientific research with data that supports the conclusions. Experiments must have been conducted rigorously, with appropriate controls, replication, and sample sizes. The conclusions must be drawn appropriately based on the data presented.

Reviewer #1: Yes

Reviewer #2: Yes

---

2. Has the statistical analysis been performed appropriately and rigorously?

Reviewer #1: No

Reviewer #2: Yes

---

3. Have the authors made all data underlying the findings in their manuscript fully available?

The PLOS Data policy requires authors to make all data underlying the findings described in their manuscript fully available without restriction, with rare exception (please refer to the Data Availability Statement in the manuscript PDF file). The data should be provided as part of the manuscript or its supporting information, or deposited to a public repository. For example, in addition to summary statistics, the data points behind means, medians and variance measures should be available. If there are restrictions on publicly sharing data—e.g. participant privacy or use of data from a third party—those must be specified.

Reviewer #1: Yes

Reviewer #2: Yes

---

4. Is the manuscript presented in an intelligible fashion and written in standard English?

PLOS ONE does not copyedit accepted manuscripts, so the language in submitted articles must be clear, correct, and unambiguous. Any typographical or grammatical errors should be corrected at revision, so please note any specific errors here.

Reviewer #1: Yes

Reviewer #2: Yes

---

5. Review Comments to the Author

Please use the space provided to explain your answers to the questions above. You may also include additional comments for the author, including concerns about dual publication, research ethics, or publication ethics. (Please upload your review as an attachment if it exceeds 20,000 characters)

Reviewer #1: The manuscript by Jadhav et al demonstrated the development and validation of a monoclonal antibody specifically recognize alternatively spliced secreted Klotho (secKL) in human plasma. The authors performed direct, indirect, and sandwich ELISA strategies to confirm the antibody recognized only the secKL but not the soluble Klotho (sKL), which is generated by proteolytic cleavage of transmembrane form of Klotho. The secKL contains a unique 15AA sequence at the C-terminal. Therefore, the authors showed that the successful generation of the 15AA-specific

monoclonal antibody that could be used to specifically target secKL in human plasma samples.

Overall, the experimental design and results of this study is straightforward. The reviewer have some comments for the authors to consider:

1. The X-axis labeling in Fig 1A and 1B is likely mislabeled if the authors intend to show Log10 value of the Ab dilution factors. Also, the scale of the Y-axis for Fig 1A, 1B, 1C, 1D, 1E, and 1F are different. Some figures used Log10 OD450nm and some used OD450nm. It is suggested to re-plot the figures with consistent scales.

We thank the reviewer for their wonderful comments. We have re-plotted all the graphs to reflect consistency in the scales.

The x-axis for Fig 1A and 1B has been updated to reflect the antibody concentrations. The scales for the Y-axis for Fig 1A,1B,1C, 1D, 1E and 1F have been re-plotted and are now consistent. All graphs now show OD450 nm on the Y-axes.

2. Similarly, the X-axis of Fig 2A is not correct.

We thank you again for your suggestion. We have re-plotted the graph for Fig 2A with OD 450nm on the Y-axis and standard concentrations on the X-axis.

3. In Fig 2B, the authors showed the serum secKL levels. What is the levels total KL (secKL+sKL) in these samples based on commercial KL kit? and what is percentage of secKL among total circulating KL?

We thank the reviewer for this insightful comment. Our ultimate goal is to be able to detect both, secKL and sKL proteins and determine their differential levels in normal Vs disease states thereby attributing their contribution to either the normal or the disease state. In order to be able to reliably do this, we first need to determine the affinities for secKL Vs sKL proteins of the commercial antibody. At this point we do not have a clear understanding of whether the antibody has a preference for either isoform and whether the two isoforms compete with each other to bind to the antibody. Lacking this information, we cannot clearly determine, in the total circulating KL that the commercial antibody detects, what fraction each isoform represents. If the two isoforms were to be competing for the antibody (which is as yet unknown), it will further cloud the contribution from each isoform and therefore might mislead the final values, especially given the fact that their levels are altered in the disease state.

4. It is not clear why the error bars are not shown in all Figures. How many experimental replicates were performed to generate the representative results? It is recommended to describe the information in figures legend or M&M section.

We thank the reviewer you for their comments.

We have re-plotted the graphs to include error bars (+/- standard deviation). In addition, each result shown in this manuscript is a representative of three replicates. This information has also been updated in the "Materials and Methods" section, Line 85-87, page 5.

"Each result is a representative of three experimental replicates. Error bars are +/- standard deviation unless indicated otherwise."

Reviewer #2: This is a clinically relevant paper that is likely to have translational relevance. Reliable antibodies that detect the secreted Klotho isoform in human plasma are required to assess it's potential as a biomarker of age-related disease. Please rearrange the paper so that the figure legends go to the end rather in the middle of the results section. Statistical analyses comparing the curves for figure 1C,D,E should be undertaken.

We thank the reviewer for their kind words on the "translational relevance" of this paper.

We apologize for the inconvenience. We have formatted the manuscript per the journal's recommended guidelines, which suggests inserting the figure legend in the text right after the first mention of the figure.

Fig 1C, 1D and 1E represent the specificity of the secKL antibody towards the secKL protein and the lack of specificity for the commercial antibody in terms of detecting both secKL and sKL proteins. While the secKL antibody is clearly able to detect the secKL protein over a wide range of dilution series, it is unable to detect the sKL protein over a similar dilution series under identical experimental conditions. This is clearly observed by the near zero OD values for the sKL protein, clearly indicating that the secKL antibody is unable to detect the sKL protein. On the contrary, the commercial antibody is able to detect both secKL and sKL proteins as seen by the non-zero OD values for both.

---

6. PLOS authors have the option to publish the peer review history of their article (what does this mean?). If published, this will include your full peer review and any attached files.

If you choose "no", your identity will remain anonymous but your review may still be made public.

Do you want your identity to be public for this peer review? For information about this choice, including consent withdrawal, please see our Privacy Policy.

Reviewer #1: No

Reviewer #2: No
